# Circulating miRNAs as a marker of metastatic disease and prognostic factor in metastatic breast cancer

**DOI:** 10.18632/oncotarget.26629

**Published:** 2019-01-29

**Authors:** Chara Papadaki, Giannis Stoupis, Leuteris Tsalikis, Alexia Monastirioti, Maria Papadaki, Neofytos Maliotis, Michalis Stratigos, Georgios Mastrostamatis, Dimitrios Mavroudis, Sofia Agelaki

**Affiliations:** ^1^ Laboratory of Translational Oncology, School of Medicine, University of Crete, Heraklion, Greece; ^2^ Department of Medical Oncology, University General Hospital, Heraklion, Crete, Greece

**Keywords:** circulating miRNAs, breast cancer, metastasis, prognosis

## Abstract

**Background:**

Circulating miRNAs (miRs) are increasingly recognized as potential biomarkers in cancer. We aimed to evaluate the differential expression of miR-23b and miR-190 which are involved in tumor dormancy, miR-21 involved in metastasis and miR-200b and miR-200c involved in epithelial-mesenchymal transition (EMT) and metastasis, in the plasma of patients with early and metastatic breast cancer (MBC). We also aimed to identify associations of the expression levels with patient and disease characteristics and outcomes in metastatic patients treated with first-line chemotherapy.

**Results:**

miR-21 (*p* < 0.001), miR-23b (*p* = 0.033), miR-200b (*p* < 0.001) and miR-200c (*p* < 0.001) expression was higher in metastatic compared to early breast cancer. ROC curve analysis showed that miR-21 (AUC = 0.722; *p* < 0.001) and miR-200b (AUC = 0.720; *p* < 0.001) distinguished with high accuracy among the two disease states, whereas the combination of miR-21, miR-190, miR-200b and miR-200c, further improved accuracy (AUC = 0.797; *p* < 0.001). High miR-200b expression independently predicted for shorter OS (*p* = 0.026) in MBC. High expression of both miR23b and miR-190 emerged as a strong independent factor associated with shorter PFS (*p* = 0.001) in *de novo* metastatic patients and high miR-200b independently predicted for decreased OS in the HER2-negative subgroup (*p* = 0.007).

**Materials and Methods:**

Blood samples were obtained from patients with early (*n* = 133) and MBC (*n* = 110) before adjuvant or first-line chemotherapy, respectively. Plasma miRNA expression levels were assessed by RT-qPCR and were classified as high or low according to the median values.

**Conclusions:**

Our results are in support of the concept that circulating miRNAs represent a tool with significant diagnostic and prognostic implications in breast cancer.

## INTRODUCTION

Despite advances in diagnosis and treatment, breast cancer remains the leading cause of cancer-related death in women worldwide [1]. Twenty to 30% of patients with early disease develop disease recurrence which remains the main cause of morbidity and mortality for these patients [2]. Unfortunately, the prognosis of patients with advanced or recurrent breast cancer has only modestly improved during the last three decades [3] with median survival and 5-year survival of approximately 3 years and 25%, respectively [4]. The outcome of metastatic patients depends on clinicopathologic factors such as hormone receptor (HR) and human epidermal growth factor receptor 2 (HER2) status, performance status, age at initial diagnosis and site and number of distant metastases [5, 6]. However, breast cancer is extremely heterogeneous with diverse clinical outcomes that cannot be captured by current prognostic factors [7]. Novel prognostic markers are needed to better stratify metastatic patients and to provide meaningful prognostic estimates [7, 8].

MicroRNAs (miRNAs) are small (22 nt) non-coding RNAs involved in the epigenetic regulation of mRNA [9]. miRNAs are dysregulated in human cancers [10] and operate as oncogenes or tumor suppressor genes, depending on the context [11]. Deregulated miRNA expression is involved in different steps of tumor progression including tumor dormancy, EMT, proliferation and metastasis [12, 13]. The potential of miRNAs as biomarkers in cancer has been increasingly recognized [14]. Indeed, recent studies reveal that miRNA expression in tumor samples has been associated with tumor aggressiveness, response to treatment and patient outcomes in various tumor types including breast cancer [12, 15–17]. Unique miRNA profiles evaluated in the serum or plasma have a role in the early detection of cancer [17, 18], in the discrimination between metastatic and non-metastatic disease states [19, 20] and in the prediction of clinical outcome in patients with cancer [21, 22]. In addition, in a recent report, a serum miRNA signature predicted response in patients with ΗΕR2-positive disease receiving the targeted therapy trastuzumab [23].

In a recent study we showed that the expression of miR-21 (related to metastasis), mir-23b and miR-190 (related to tumor dormancy) and miR-200b/c (related to EMT), evaluated in the plasma of patients with early breast cancer before the initiation of adjuvant chemotherapy were differentially expressed among patients who subsequently experienced disease recurrence compared to those who remained disease-free during follow-up [24]. Interestingly, miRNAs could predict for disease recurrence years before the clinical detection of metastases [24].

Based on the above findings we sought to evaluate whether the aforementioned miRNAs could also discriminate among patients with early and MBC and whether they could be used for the refinement of prognosis in patients with metastatic disease.

## RESULTS

### Differential expression of miRNAs and their predictive capability in distinguishing early from MBC

We compared the expression of miRNAs in patients with early (*n* = 133) and metastatic (*n* = 70) breast cancer. The characteristics of patients with metastatic disease are presented in Table 1. Median age was 63 years (range, 30-84), 45 (64%) had *de novo* metastatic disease and 51 (73%) were HER2-negative.

**Table 1:** Characteristics of patients with MBC

Characteristic	All patients		*de novo* metastatic		HER2-negative	
	*N*	%	*N*	%	No.	%
**Patients enrolled**	70	100	45	64	51	73
**Age (years)**						
Median (range)	63 (30–84)		60 (31–82)		63 (30–84)	
**Menopausal status**						
Pre	38	54	25	56	27	53
Post	32	46	20	44	24	47
**Performance status**						
0–1	58	83	33	73	43	84
2	12	17	12	27	8	16
**Disease status at diagnosis**						
Recurrent	25	36			22	43
*de novo* metastatic	45	64			29	57
**Histological Grade**						
I/II	32	46	24	53	23	45
III	26	37	13	29	17	33
Unknown	12	17	8	18	11	22
**ER status**						
Positive	53	76	35	78	41	80
Negative	15	21	10	22	8	16
Unknown	2	3			2	4
**PR status**						
Positive	48	68	29	64	36	70
Negative	20	29	16	36	13	26
Unknown	2	3			2	4
**HER2 status**						
Positive	19	27	16	36		
Negative	51	73	29	64		
**First line chemotherapy**						
Taxane-based	45	64	28	62	33	65
Taxanes+Anthracyclines	13	19	8	18	13	25
Anthracycline-based	3	4	2	4	3	6
Others	9	13	7	16	2	4
**Response to treatment**						
CR+PR	31	44	20	44	23	45
SD+PD	39	56	25	56	28	55
**Visceral metastases**						
Yes	47	67	32	71	31	61
No	19	27	11	24	18	35
Unknown	4	6	2	4	2	4
**Non visceral metastases**						
Yes	56	80	39	87	41	80
No	10	14	4	9	8	16
Unknown	4	6	2	4	2	4

ER, estrogen receptor; PR, progesterone receptor; HER2, human epidermal growth factor receptor 2; CR, complete response; PR, partial response; SD, stable disease; PD, progressive disease.

**Figure 1:**
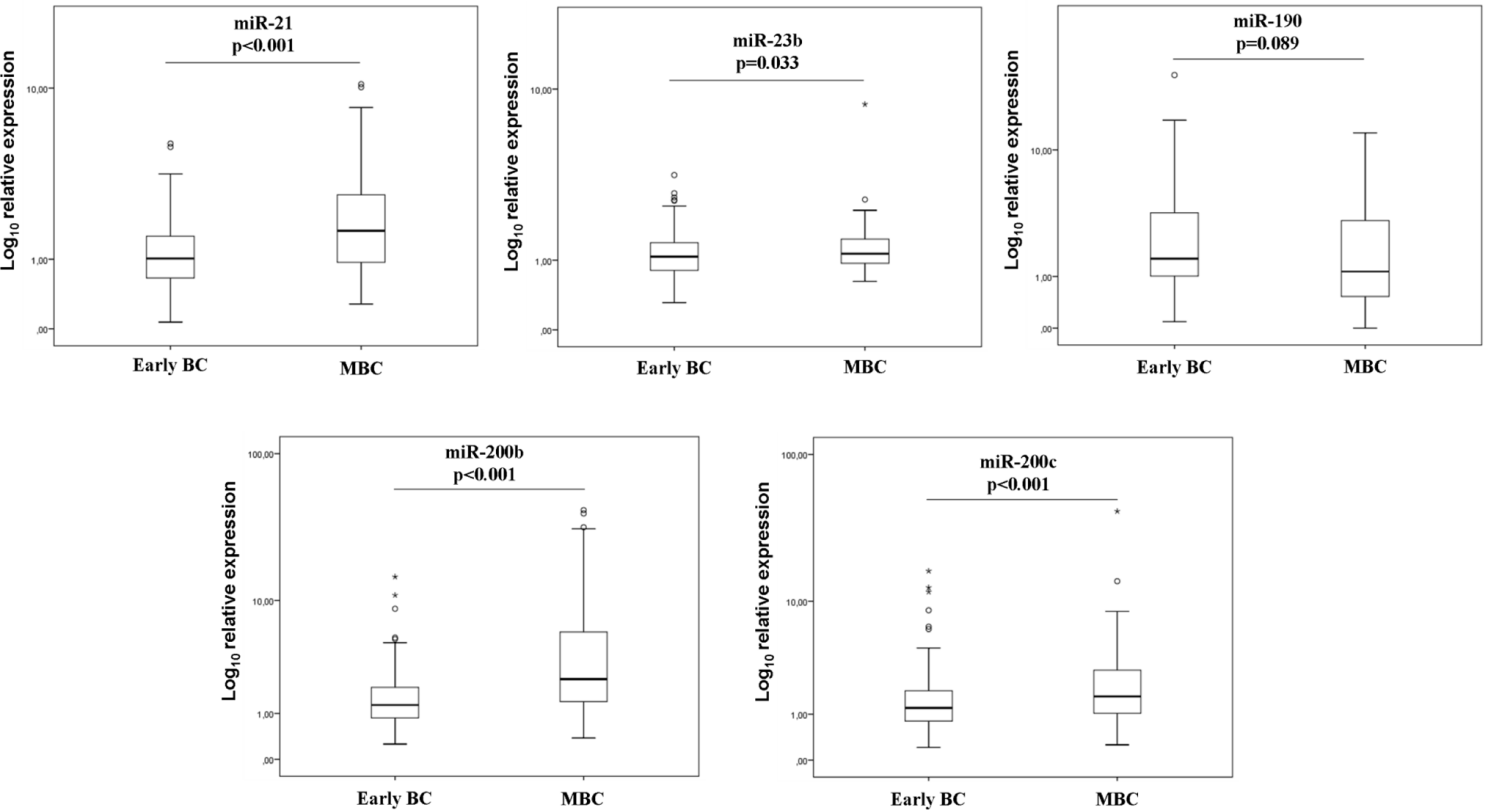
Relative expression levels of circulating miRNAs of early and metastatic patients Expression levels of miR-21 (**A**), miR-23 (**B**), miR-190 (**C**), miR-200b (**D**) and miR-200c (**E**) were evaluated in the plasma by RT-qPCR and assessed by 2^-ΔΔCt^ method. Statistically significant differences were determined using Mann-Whitney tests and the results were displayed on box plots. Horizontal line depicts median, whereas the length of the boxes is the interquartile range that represents values between the 75th and 25th percentiles of individual fold change expression values. Relative expression values on y-axis are plotted on a log_10_ scale. Circles represent outliers, whereas asterisks represent extreme outliers. *P* values are shown.

The Mann–Whitney tests for miRNA expression revealed that the expression levels of miR-21 (*p* < 0.001), miR-23b (*p* = 0.033), miR-200b (*p* < 0.001) and miR-200c (*p* < 0.001) were higher in metastatic compared to patients with early disease (Figure 1).

**Figure 2:**
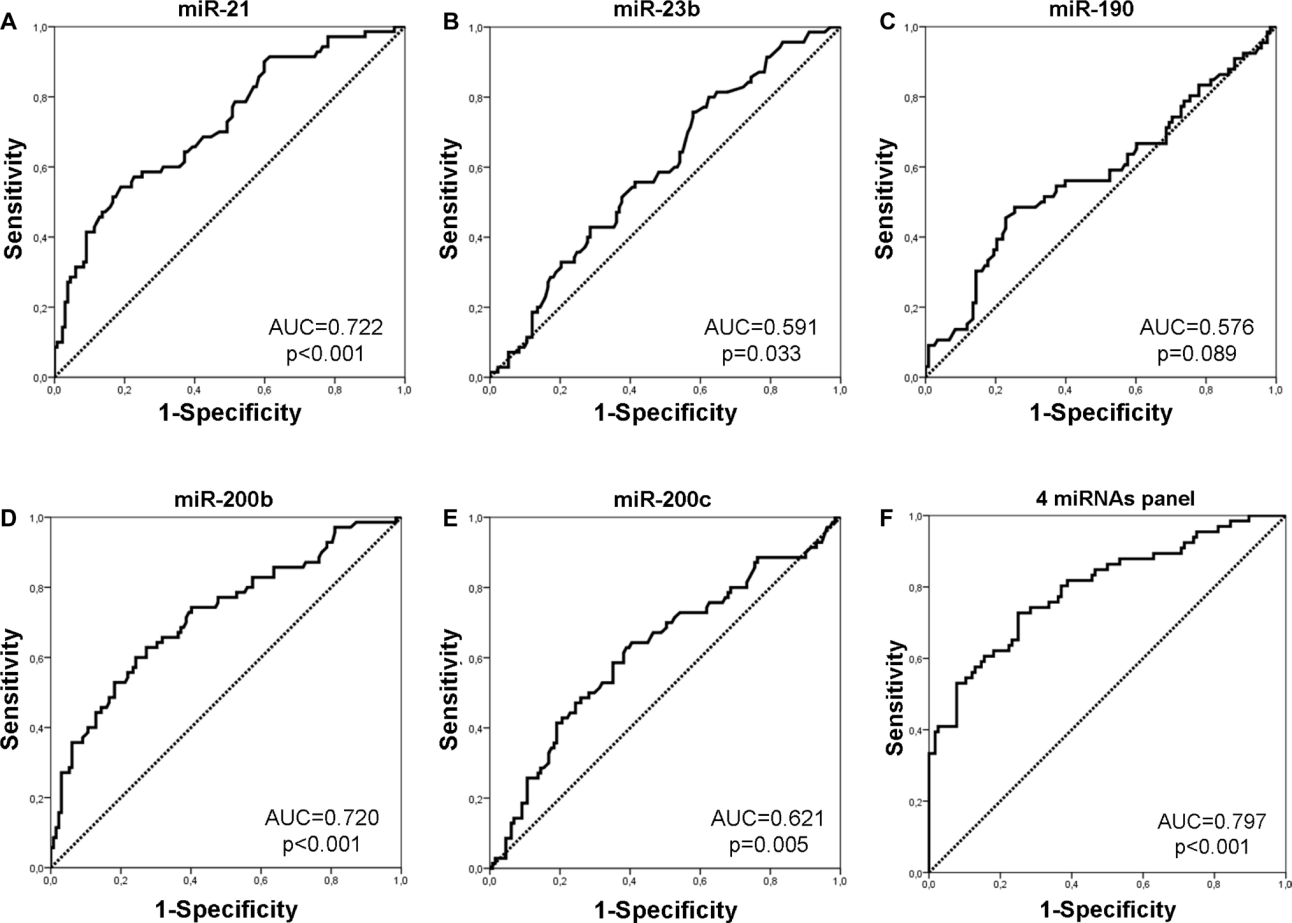
ROC curve analysis Performance of plasma miR-21 (**A**), miR-23 (**B**), miR-190 (**C**), miR-200b (**D**) and miR-200c (**E**) and their combined expression (**F**) to discriminate patients with early and those with MBC. AUC and *p* values are shown.

We next evaluated the predictive capability of plasma miRNAs in distinguishing between early and metastatic patients. Binary logistic regression incorporating various combinations of miRNAs was used and ROC curves were constructed to determine the specificity and sensitivity of miRNA expression (Figure 2 and Table 2). ROC curve analysis showed that among the investigated miRNAs, miR-21 and miR-200b expression had the highest performance with an AUC of 0.722 [sensitivity of 51.4% and specificity of 83.3% (*p* < 0.001; 95% CI 0.648–0.796)] and AUC of 0.720 [sensitivity of 60% and specificity of 75.8% (*p* < 0.001; 95%CI: 0.644–0.796)] (Figure 2A–2E). By assessing combinations of miRNAs, binary logistic regression analysis demonstrated that the panel of miR-21, miR-190, miR-200b and miR-200c had the highest predictive accuracy. Specifically, the combined ROC curve of the panel had an AUC of 0.797 with sensitivity of 72.7% and specificity of 75% (*p* < 0.001; 93% CI: 0.727-0.866) (Figure 2F).

**Table 2:** Performance of miRNAs and their combinations to predict disease status in breast cancer

Potential predictors	Cut-off value	Sensitivity %	Specificity %	AUC (95% CI)	*P*
miR-21	1.65	51.4	83.3	0.722 (0.648–0.796)	<0.001
miR-23b	0.93	75.7	42.1	0.591 (0.511–0.672)	0.033
miR-190	1.02	48.5	74.6	0.576 (0.486–0.665)	0.089
miR-200b	2.06	60.0	75.8	0.720 (0.644–0.796)	<0.001
miR-200c	1.35	62.9	61.1	0.621 (0.538–0.705)	0.005
4 miRNAs panel (miR-21, miR-190, miR-200b, miR-200c)	0.31	72.7	75	0.797 (0.727–0.866)	<0.001

AUC, area under the receiver operating curve.

### miRNA expression and statistical correlations in MBC patients

A strong correlation was observed between the expression of miR-200b and miR-200c (Spearman’s Rho: 0.763; *p* < 0.001). Moreover, a strong correlation was observed between the expression of miR-21 and miR-200c (Spearman’s Rho: 0.605; *p* < 0.001) and between miR-21 and miR-200b (Spearman’s Rho: 0.554; *p* < 0.001). A weaker but still significant correlation was revealed between miR-21 and miR-23b (Spearman’s Rho: 0.309; *p* = 0.009), between miR-23b and miR-190 (Spearman’s Rho: 0.302; *p* = 0.014) and between miR-23b and miR-200c (Spearman’s Rho: 275; *p* = 0.021) as well (Table 3).

**Table 3:** Correlation of coefficient among 5 miRNAs

	miR-21	miR-23b	miR-190	miR-200b	miR-200c
miR-21	1.000				
miR-23b	0.309^**^	1.000			
miR-190	0.136	0.302^*^	1.000		
miR-200b	0.554^**^	0.206	0.041	1.000	
miR-200c	0.605^**^	0.275^*^	0.056	0.763^**^	1.000

^**^p < 0.01; ^*^p < 0.05.

Higher miR-21 and miR-200b expression was observed in patients with pre-menopausal compared to patients with post-menopausal status (chi-squared test: 69% vs 31%; *p* = 0.015 and 67 vs 33; *p* = 0.013, respectively). Patients with low expression of miR-190 had increased incidence of bone metastases as compared to those with high expression (chi-squared test: 70% vs 30%; *p* = 0.019). We did not observe any differences in miRNA expression among patients presenting with *de novo* metastatic (*n* = 45) and those with recurrent disease (*n* = 25; Mann–Whitney tests, *p* > 0.05). Moreover, no differences in miRNA expression were revealed among HER-2 negative (*n* = 51) and HER-2 positive (*n* = 19) patients (Mann-Whitney tests, *p*>0.05).

No other significant correlations were observed between miRNA expression and clinicopathological parameters or the type of first line chemotherapy regimens administered.

### miRNA expression and clinical outcome in MBC

**Figure 3:**
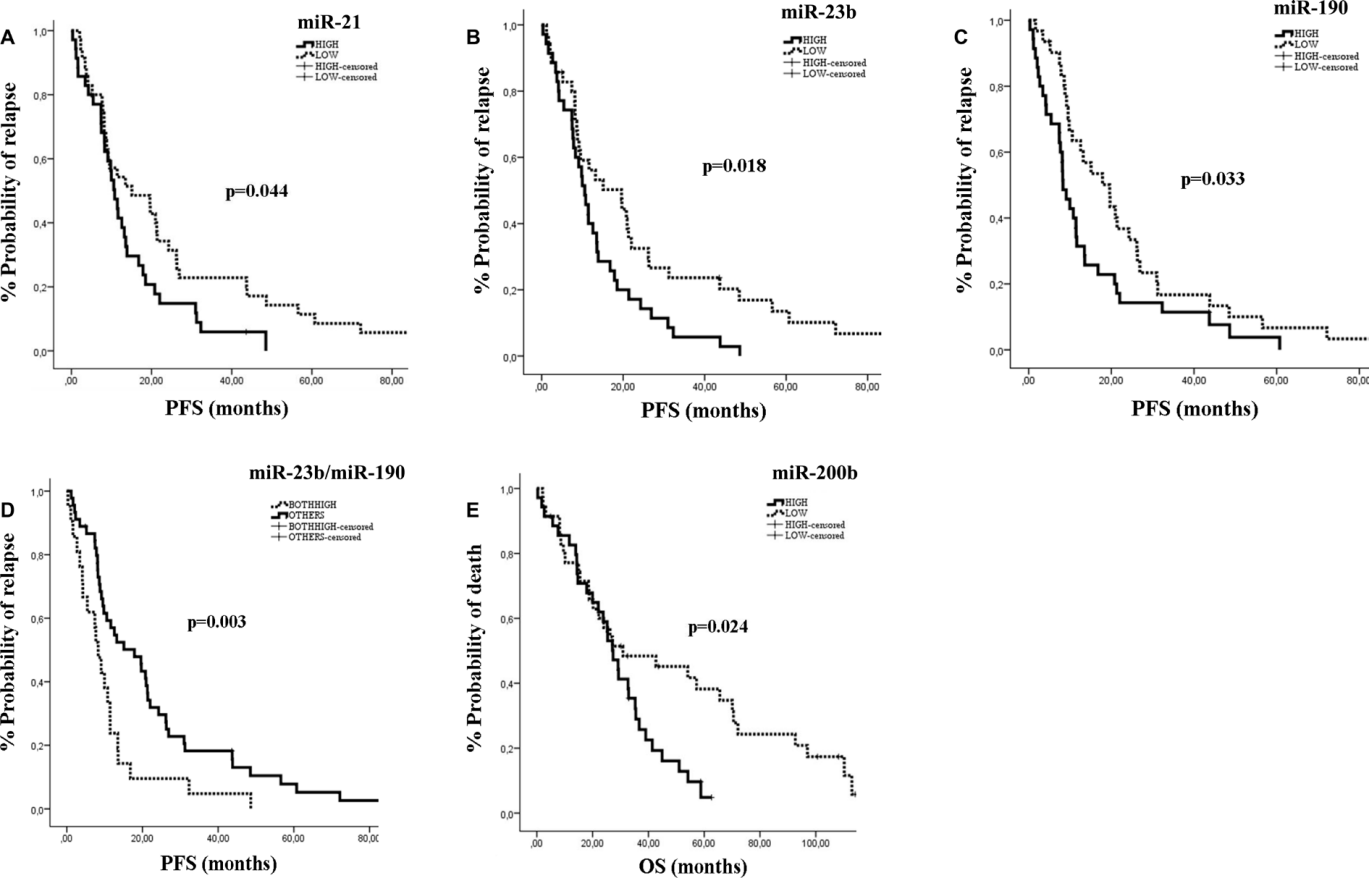
Kaplan–Meier analysis for PFS and OS according to the expression of circulating miRs in MBC patients Progression free survival and overall survival in patients with high or low miR-21 (**A**), miR-23b (**B**), miR-190 (**C**), miR-23b/miR-190 (**D**) and miR-200b (**E**). Curves were compared using the log rank test. *P* values are shown.

The median PFS and OS for the whole group of patients were 11.47 months (95% CI: 7.89-15.05) and 27.33 months (95% CI: 20.97-33.69), respectively. The type of first line chemotherapy was not associated with patients’ outcomes. We used the median expression levels for each miRNA to classify patients into high or low expression groups. Kaplan Meier survival curves demonstrated that patients with high miR-21, miR-23b or miR-190 had significantly shorter PFS compared to those with low expression (10.8 vs 15.1 months; *p* = 0.044, 10.57 vs 19.60 months; *p* = 0.018 and 8.3 vs 19.6 months; *p* = 0.033, respectively) (Figure 3A–3C). Moreover, the combination of miR-23b and miR-190 high expression was associated with shorter PFS as compared to low expression (8.3 vs 17.87 months; *p* = 0.003; Figure 3D). In addition, patients with high miR-200b had shorter OS compared to those with low expression (27.33 vs 30.87 months; *p* = 0.024 (Figure 3E). No differences in either PFS or OS were observed according to the median expression values for the remaining miRNAs.

Cox univariate analysis revealed that pre-menopausal status and recurrent disease were associated with shorter PFS (*p* = 0.017 and *p* = 0.012, respectively; Table 4), whereas recurrent disease was associated with shorter OS (*p* = 0.003). Regarding the expression of miRNAs, high miR-21, miR-23b or miR-190 and high expression of both miR-23b and miR-190 were associated with shorter PFS (*p* = 0.046, *p* = 0.02, *p* = 0.035 and *p* = 0.007, respectively; Table 4), whereas high expression of miR-200b was associated with shorter OS (*p* = 0.027; Table 4). Multivariate analysis confirmed pre-menopausal status and both miR-23b/miR-190 high expression as independent predictors for shorter PFS (*p* = 0.047 and *p* = 0.009, respectively; Table 4). In addition, disease recurrence and high expression of miR-200b were also independently associated with shorter OS (*p* = 0.003 and 0.026, respectively; Table 4).

**Table 4:** Univariate and multivariate analysis for PFS and OS in MBC (*n* = 70) patients

Univariate analysis				
Cox regression	PFS		OS	
	HR (95% CI)	*p*-value	HR (95% CI)	*p*-value
Age (<63 vs ≥63)	1.337 (0.806–2.218)	0.261	1.184 (0.707–1.980)	0,521
Menopausal status (pre vs post)	1.908 (1.120–3.250)	0.017^*^	1.628 (0.964–2.923)	0.067
PS (2-3 vs 0-1)	1.788 (0.943–3.393)	0.075	1.802 (0.940–3.454)	0.076
Disease status (recurrent vs *de novo*)	1.943 (1.157–3.262)	0.012^*^	2.231 (1.306–3.814)	0.003^*^
Grade (III vs I/II)	1.203 (0.701–2.065)	0.503	1.232 (0.703–2.159)	0.465
ER status (negative vs positive)	1.600 (0.879–2.911)	0.124	1.600 (0.879–2.911)	0.124
PR status (negative vs positive)	1.151 (0.671–1.974)	0.609	1.151 (0.671–1.974)	0.609
HER2 (positive vs negative)	1.254 (0.732–2.148)	0.410	1.254 (0.732–2.148)	0.410
Visceral metastases (no vs yes)	1.002 (0.568–1.766)	0.996	1.002 (0.568–1.766)	0.996
Non-visceral metastases (yes vs no)	1.268 (0.623–2.584)	0.513	1.268 (0.623–2.584)	0.513
Bone metastases (no vs yes)	1.321(0.798–2.187)	0.278	1.083 (0.634–1.849)	0.771
miR-21 (high vs low)	1.680 (1.009–2.799)	0.046^*^	1.589 (0.916–2.756)	0.100
miR-23b (high vs low)	1.828 (1.101–3.035)	0.020^*^	1.299 (0.772–2.186)	0.324
miR-190 (high vs low)	1.719 (1.038–2.846)	0.035^*^	1.143 (0.677–1.928)	0.617
miR-200b (high vs low)	1.396 (0.834–2.336)	0.204	1.893 (1.076–3.331)	0.027^*^
miR-200c (high vs low)	1.229 (0.752–2.010)	0.411	1.096 (0.658–1.025)	0.725
miR-23b/190 (both high vs others)	2.107 (1.225–3.623)	0.007^*^	1.362 (0.779–2.382)	0.278
**Multivariate analysis**				
Menopausal status (pre vs post)	1.724 (1.006–2.953)	0.047^*^	–	–
Disease status (recurrent vs *de novo*)	–	–	2.249 (1.312–3.857)	0.003^*^
miR-200b (high vs low)	–	–	1.916 (1.082–3.395)	0.026^*^
miR-23b/190 (both high vs others)	2.054 (1.195–3.530)	0.009^*^		

PFS, progression free survival; OS, overall survival; ER, estrogen receptor; PR, progesterone receptor; HER2, human epidermal growth factor receptor 2; CR, complete response; PR, partial response; SD, stable disease; PD, progressive disease; ^*^p < 0.05.

### miRNA expression and clinical outcome according to patient subgroups

#### Patients with *de novo* metastatic disease

Patients’ characteristics are shown in Table 1. Higher miR-21 expression was observed in pre-menopausal as compared to post-menopausal and in HER2-negative as compared to HER2-positive patients (chi-squared test: 74% vs 26%; *p* = 0.012 and 78% vs 22%; *p* = 0.047, respectively). Moreover, lower miR-190 expression was observed in patients with bone metastases as compared to patients without bone metastases (chi-squared test: 60% vs 40%; *p* = 0.008).

**Figure 4:**
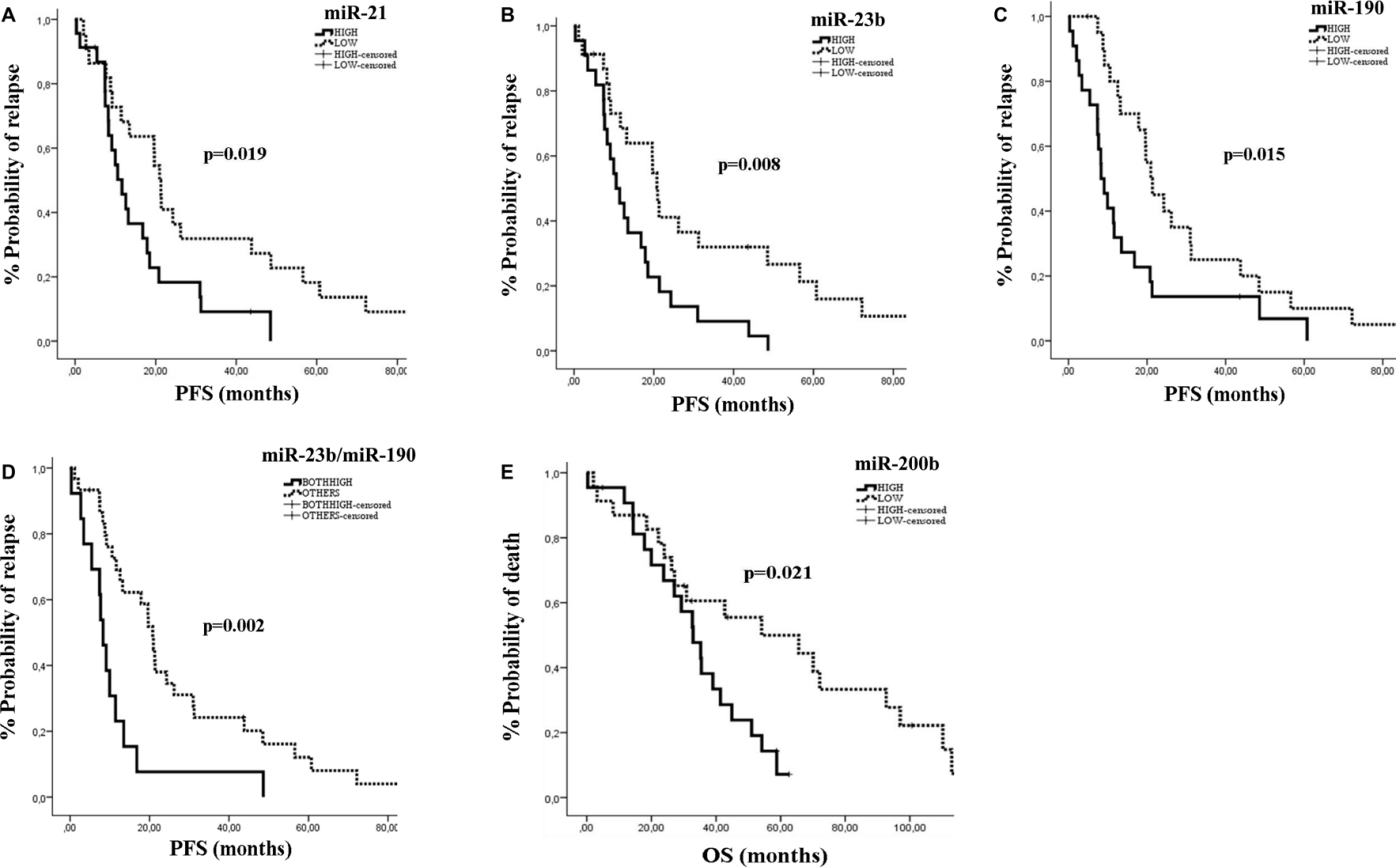
Kaplan–Meier analysis for PFS and OS according to the expression of circulating miRNAs in *de novo* metastatic patients Progression free survival and overall survival in patients with high or low miR-21 (**A**), miR-23b (**B**), miR-190 (**C**), miR-23b/miR-190 (**D**) and miR-200b (**E**). Curves were compared using the log rank test. *P* values are shown.

The median PFS and OS for the *de novo* metastatic subgroup was 16.8 months (95% CI: 9.38–24.15) and 35.47 months (95% CI: 24.92–46.02) respectively. Patients with miR-21, miR-23b or miR-190 high expression had significantly shorter PFS compared to patients with low expression (11.63 vs 20.97 months; *p* = 0.019, 10.57 vs 20.97; *p* = 0.008, and 8.3 vs 20.97 months; *p* = 0.015, respectively) (Figure 4A–4C). Furthermore, patients with both miR-23b and miR-190 high expression had shorter PFS (8.3 vs 20.8 months; *p* = 0.002; Figure 4D). High miR-200b was significantly associated with worse OS (32.9 vs 54.1 months; *p* = 0.021) compared to patients with low expression (Figure 4E).

In univariate analysis, pre-menopausal status and poor performance status were associated with significantly shorter PFS (*p* = 0.016 and *p* = 0.017, respectively) and OS (*p* = 0.025 and *p* = 0.003, respectively) (Table 5). Also, high expression of miR-21, miR-23b or miR-190 were significantly associated with shorter PFS (*p* = 0.016, *p* = 0.017 and *p* = 0.028, respectively). In addition, both miR-23b and miR-190 high expression were also associated with shorter PFS (*p* = 0.003; Table 5). In multivariate analysis, pre-menopausal status, poor performance status, and both miR-23b and miR-190 high expression were independent prognostic factors for decreased PFS (*p* < 0.001, *p* = 0.001 and *p* = 0.001, respectively), whereas only pre-menopausal status emerged as independent predictor for decreased OS (*p* < 0.001; Table 5).

**Table 5:** Univariate and multivariate analysis for PFS and OS in *de novo* metastatic (*n* = 45) and *HER2-negative* (*n* = 51) patients

Univariate analysis				
Cox regression	PFS		OS	
	HR (95% CI)	*p*-value	HR (95% CI)	*p*-value
***de novo* metastatic**				
Age (<60 vs ≥60)	1.633 (0.859–3.105)	0.135	1.099 (0.561–2.152)	0.784
Menopausal status (pre vs post)	2.420 (1.181–4.957)	0.016^*^	2.402 (1.118–5.161)	0.025^*^
PS (2-3 vs 0-1)	2.364 (1.167–4.789)	0.017^*^	3.185 (1.503–6.752)	0.003
Grade (III vs I/II)	1.047 (0.509–2.154)	0.901	1.112 (0.524–2.357)	0.782
ER status (negative vs positive)	1.229 (0.586–2.580)	0.585	1.388 (0.629–3.062)	0.417
PR status (negative vs positive)	1.223 (0.646–2.315)	0.536	1.215 (0.609–2.425)	0.581
HER2 (positive vs negative)	1.465 (0.778–2.758)	0.237	1.049 (0.528–2.084)	0.891
Visceral metastases (yes vs no)	1.138 (0.549–2.361)	0.728	1.480 (0.704–3.112)	0.301
Non-visceral metastases (yes vs no)	1.668 (0.511–5.439)	0.396	1.846 (0.552–6.174)	0.320
Bone metastases (yes vs no)	1.235 (0.646–2.361)	0.524	1.889 (0.897–3.979)	0.094
miR-21 (high vs low)	2.257 (1.164–4.376)	0.016^*^	1.796 (0.872–3.698)	0.112
miR-23b (high vs low)	2.200 (1.150–4.211)	0.017^*^	1.897 (0.954–3.770)	0.068
miR-190 (high vs low)	2.023 (1.078–3.794)	0.028^*^	1.080 (0.559–2.083)	0.819
miR-200b (high vs low)	1.767 (0.911–3.428)	0.092	2.359 (1.111–5.007)	0.025
miR-200c (high vs low)	1.598 (0.85602.982)	0.141	1.022 (0.534–1.954)	0.948
miR-23b/mir-190 (both high vs others)	2.857 (1.415–5.767)	0.003^*^	1.477 (0.729–2.992)	0.279
**Multvariate analysis**				
Menopausal status (pre vs post)	4.125 (1.871–9.091)	<0.001^*^	5.658 (2.485–12.879)	<0.001^*^
PS (2-3 vs 0-1)	3.773 (1.754–8.130)	0.001^*^		
miR-23b/miR-190	3.670 (1.713–7.867)	0.001^*^		
***HER2-negative***				
**Univariate analysis**				
Age (<63 vs ≥63)	1.280 (0.700–2.341)	0.423	1.145 (0.625–2.098)	0.661
Menopausal status (pre vs post)	1.768 (0.962–3.247)	0.066	1.698 (0.906–3.179)	0.098
Performanc status (2-3 vs 0-1)	1.659 (0.763–3.607)	0.202	1.388 (0.635–3.034)	0.411
Disease status (recurrent vs *de novo*)	2.059 (1.132–3.747)	0.018^*^	2.379 (1.296–4.369)	0.005^*^
Grade (III vs I/II)	1.077 (0.556–2.087)	0.826	1.252 (0.634–2.473)	0.517
ER status (negative vs positive)	1.593 (0.701–3.622)	0.266	1.102 (0.462–2.631)	0.826
PR status (negative vs positive)	1.197 (0.614–2.334)	0.597	1.145 (0.572–2.290)	0.702
Visceral metastases (yes vs no)	1.002 (0.537–1.868)	0.996	1.060 (0.557–2.018)	0.859
Non-visceral metastases (yes vs no)	1.159 (0.512–2.622)	0.723	1.181 (0.520–2.686)	0.691
Bone metastases (yes vs no)	1.632 (0.900–2.958)	0.107	1.511 (0.815–2.800)	0.190
miR-21 (high vs low)	1.691 (0.927–3.084)	0.087	1.533 (0.807–2.914)	0.192
miR-23b (high vs low)	1.715 (0.952–3.088)	0.072	1.336 (0.734–2.432)	0.343
miR-190 (high vs low)	1.571 (0.860–2.870)	0.142	1.497 (0.795–2.820)	0.211
miR-200b (high vs low)	1.346 (0.739–2.450)	0.331	2.342 (1.214–4.518)	0.011^*^
miR-200c (high vs low)	1.169 (0.654–2.091)	0.598	1.333 (0.728–2.440)	0.352
miR-21/miR-23b (both high vs others)	1.981 (1.053–3.725)	0.034^*^	1.962 (1.039–3.707)	0.038^*^
**Multvariate analysis**				
Disease status (recurrent vs *de novo*)	2.059 (1.132–3.747)	0.018^*^	2.543 (1.372–4.713)	0.003^*^
miR-200b (high vs low)			2.531 (1.291–4.962)	0.007^*^

PFS, progression free survival; OS, overall survival; ER, estrogen receptor; PR, progesterone receptor; HER2, human epidermal growth factor receptor 2; CR, complete response; PR, partial response; SD, stable disease; PD, progression disease; ^*^p < 0.05.

### Her-2 negative patients

Patients’ characteristics are shown in Table 1. Higher miR-23b expression was observed in PR-positive as compared to PR- negative patients (chi-squared test: 85% vs 15%; *p* = 0.041). Furthermore, patients with low miR-190 expression had increased risk of bone metastases as compared to patients with high expression.

**Figure 5:**
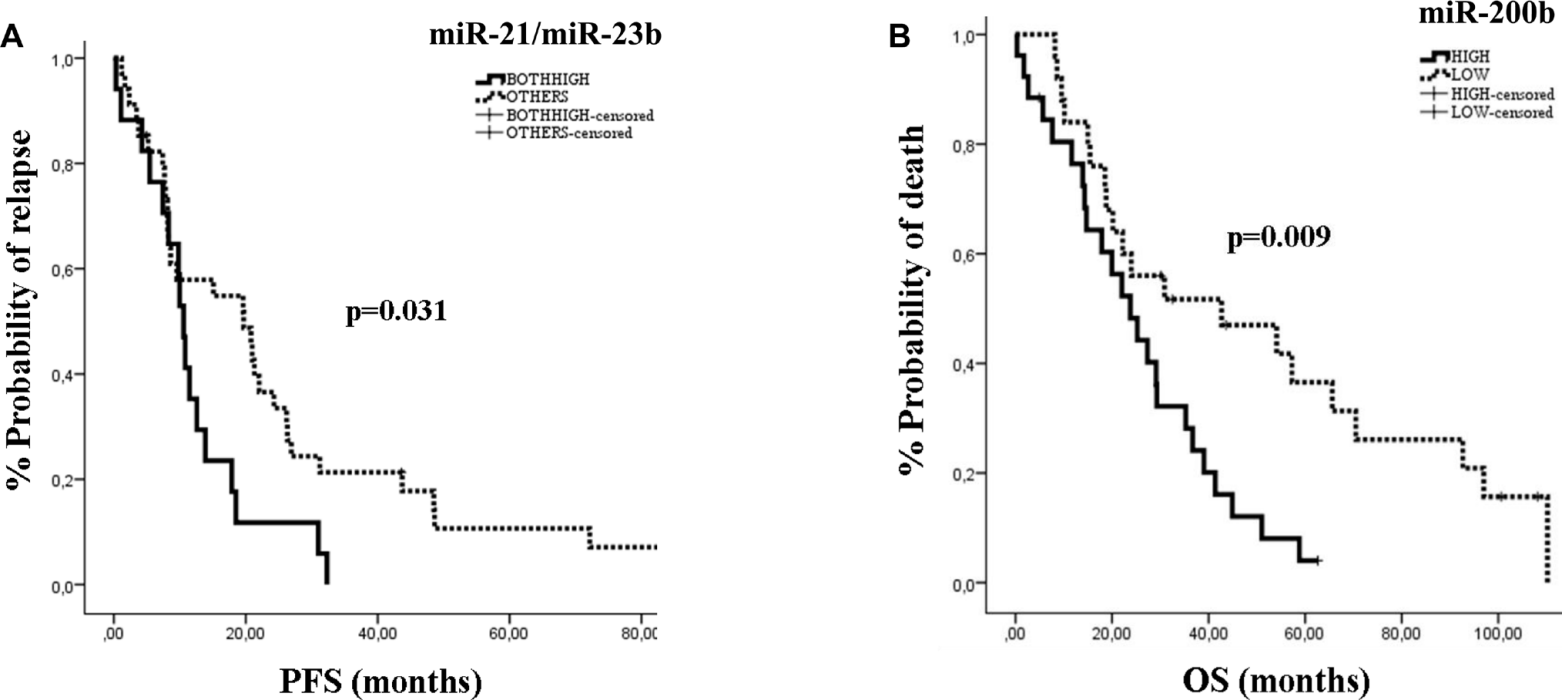
Kaplan–Meier analysis for PFS and OS according to the expression of circulating miRNAs in HER2-negative patients Progression free survival according to the combined expression of miR-21/miR-23b (**A**) and overall survival according to the expression miR-200b (**B**). Curves were compared using the log rank test. *P* values are shown.

The median PFS and OS were 12.57 months (95% CI: 6.672-18.47) and 27.33 months (95% CI: 19.18-35.48), respectively in this subgroup of patients. Patients with both miR-21 and miR-23b high expression had significantly shorter PFS (10.57 vs 19.67 months; *p* = 0.031) compared to patients with low expression (Figure 5A). Furthermore, patients with miR-200b high expression had shorter OS compared to patients with low expression (23.8 vs 42.7 months; *p* = 0.009) (Figure 5B).

Cox univariate analysis revealed that recurrent breast cancer and high expression of both miR-21 and miR-23b were associated with shorter PFS (*p* = 0.018, and *p* = 0.034, respectively) (Table 5). Moreover, recurrent disease and high expression of miR-200b and both miR21 and miR-23b high expression were associated with shorter OS (*p* = 0.005, *p* = 0.011 and *p* = 0.038, respectively) (Table 5). In multivariate analysis, recurrent disease was independently associated with worse PFS (*p* = 0.018) and worse OS (*p* = 0.003), whereas, miR-200b high emerged as an independent prognostic factor for worse OS (*p* = 0.007) (Table 5).

## DISCUSSION

MBC has a dismal prognosis and novel biomarkers indicative of the inherent biologic behaviour of the disease could improve patient prognostication. The identification of novel blood-based biomarkers with clinical application in breast cancer represents a challenge in translational cancer research. In the current study we report that the dormancy and metastasis-related miR-21, miR-23b, miR-200b and miR-200c are differentially expressed among patients with early and MBC and that the combination of miR-21, miR-190, miR-200b and miR-200c was more informative in predicting early versus metastatic disease status. Furthermore, we show that the expression levels of circulating miRNAs are correlated with patient and disease characteristics and independently predict for clinical outcome in metastatic patients treated with first-line chemotherapy.

In our previous work, plasma expression levels of miR-21, miR-23b, miR-190 and miR-200b/c differed among patients with early breast cancer who subsequently recurred and those who remained disease-free during follow-up [24]. Interestingly, herein we show that the same miRs, except for the dormancy-related miR-190, were also differentially expressed among patients with early and MBC. However, miR-190 was still included in the panel of the four miRs with the highest accuracy in predicting metastatic disease stage.

Differential miRNA expression in body fluids has been previously reported among early and MBC [19, 20, 25] and suggests that miRNAs may be linked to a particular biology of breast carcinomas favoring metastatic progression. Our results suggest that similar gene regulatory networks underlie the complex interactions between tumor cells and cells of the tumor microenvironment [26] during both the subclinical and the clinically evident phase of the metastatic procedure. Moreover, they complement previous observations that these interactions can be uncovered in the circulation through the evaluation of unique miRNA profiles [27].

It has been consistently demonstrated that miRNA expression in tumor tissue is correlated with clinical and histopathological characteristics in breast cancer [28]. Here we demonstrate, for the first-time, higher plasma miR-21 and miR-200b expression in pre-menopausal compared to post-menopausal patients with metastatic disease. Although up-regulated miR-21 expression has been reported in ER-positive, in HER2-positive or in ER-negative breast cancer tissues [29, 30], in accordance to Jurkovicova *et al.* [25] we found no association between plasma miR-21 expression and hormone receptor or HER2 status. miR-200 family members are up-regulated by estradiol/estrogen signaling [31], whereas, miR-190 has been reported as the highest up-regulated miRNA in hormone-dependent breast cancers [32]. However in our cohort, no associations were revealed between miR-200 or miR-190 expression levels and hormone receptor status. On the other hand, we showed an association between low miR-190 and increased incidence of bone metastases. miR-190 is found among the miRNAs with multiple binding sites on gene targets known to be important for osteoclast differentiation and function, which is of vital importance for the development of bone metastases [33]. The herein reported association of low miR-190 levels with bone metastases suggests that miR-190 should be further investigated as a marker for organotropic metastasis in breast cancer. Moreover, our observation suggests that miRNAs involved in osteoclastogenesis may potentially serve as biomarkers for bone metastasis development.

The miR-200 family has conflicting roles in metastatic progression [34]. Interestingly, miRNAs of the miR-200 family are secreted in extracellular vesicles from metastatic mouse and human breast cancer cell lines to promote cancer cell metastasis [35]. In agreement with the pro-metastatic role of the miR-200 family, clinical studies demonstrate that miR-200b and miR-200c expression is increased in the plasma of metastatic patients and is correlated with poor outcomes [36–38]. We have previously shown that miR-200b and miR-200c expression was higher in relapsed compared to non-relapsed patients with early breast cancer [24] and herein we demonstrate that metastatic patients harbour higher levels of miR-200b and miR-200c compared to early breast cancer patients. In addition, miR-200b discriminated with high accuracy among patients with early and metastatic disease, whereas miR-200b high expression also emerged as independent predictor of poor survival both in the whole group as well as in the HER2-negative subgroup of metastatic patients.

Although miR-23b was identified as a dormancy-related miRNA in a bone marrow-metastatic human breast cancer cell line [39] and inhibited proliferation [40], cell migration and invasion in glioblastoma cells [41], in other studies miR-23b was correlated with metastasis and breast cancer progression. In clinical studies, miR-23b/27b/24 expression was higher in cancerous compared to normal tissues and was associated with poor outcome in breast cancer [42], thus supporting our observations regarding the association between high miR-23b and lower PFS in metastatic patients. Importantly, the combination of high miR-23b and miR-190 also emerged as an independent predictor for worse PFS. In contrast, we previously demonstrated that patients with early breast cancer who relapsed had lower miR-190 expression levels compared to non-relapsed patients [24] and accordingly, Yu *et al.*, showed that patients with early disease and high miR-190 expression in cancer tissue had a significantly better DFS and OS compared to those with low expression [43]. Our observations suggest a differential function of miR-190 possibly related to the specific disease context. It has been suggested that various miRNAs could produce tumor suppressive or oncogenic effects as a result of the suppression of both tumor suppressive and oncogenic mRNAs and it is the balance between the multiple processes during carcinogenesis and tumor progression that ultimately determines the net function of a specific miRNA [11]. Patients with recurrent disease possibly represent a different prognostic group compared to those with *de novo* metastatic disease, although the issue remains controversial [44]. In our cohort, recurrent breast cancer was independently associated with decreased OS, both in the whole group as well as in the HER2-negative subgroup of metastatic patients. When we investigated potential markers associated with outcome in patients with *de novo* metastatic disease, we found that premenopausal status and performance status independently predicted for worse PFS, whereas, the miR-23b/miR-190 high also emerged as an independent predictor for worse PFS.

MiR-21 has been extensively studied as an oncogenic miRNA that promotes cell growth, invasion and tumor metastasis through the inhibition of tumor suppressor genes [45]. Clinical evidence indicates that miR-21 is upregulated in breast cancer tissue and is correlated with advanced stages of disease, metastasis and poor prognosis in breast cancer [46, 47]. Furthermore, several reports demonstrate the potential of circulating miR-21 as a marker for the detection of breast cancer [48, 49]. In our previous report, miR-21 expression levels could discriminate among relapsed and non-relapsed patients with early breast cancer [24] and herein, miR-21 expression distinguished with high accuracy patients with early from patients with metastatic disease. These and other observations suggest that circulating miR-21 could also have a role as a marker for the diagnosis of metastasis in breast cancer [50]. Interestingly, although high miR-21 expression was associated with lower PFS in the whole group and in *de novo* metastatic patients, it does not add further diagnostic value regarding the aggressiveness of metastatic disease in these patients, since miR-21 expression was correlated with pre-menopausal status that independently predicted for shorter PFS.

It is increasingly recognized that dynamic changes in miRNA expression profiles are generated during tumor initiation and metastatic progression and many studies have focused on analyzing circulating miRNAs to determine their potential as biomarkers [51]. However, different biological and technical factors can influence the expression profiles of circulating miRNAs [52] and in the present study, we considered pre-analytical and analytical parameters very carefully, taking into account the variables that could lead to bias in miRNA quantification [53, 54].

Currently, only a few studies have identified circulating miRNAs associated with prognosis in patients with MBC and although our results are promising, some limitations exist and should be addressed in future studies. Due to the relatively small sample size, our results should be further validated in a larger study to allow for more robust statistical associations. The higher number of study patients would allow for firm conclusions to be drawn regarding patient subgroups for which different miRNAs represent potential prognostic biomarkers. There is as yet no conclusive evidence on the clinical utility of miRNAs, however, the data presented in this manuscript, as well as in other reports, suggest that circulating miRNAs represent non-invasive biomarkers to be used not only in the detection of breast cancer, but also in the prediction of metastasis and disease outcome.

In summary, the results of this study demonstrate that a panel of four miRNAs, namely, miR-21, miR-190, miR-200b and miR-200c can discriminate between early and MBC. Moreover, it is shown that miRNA expression can independently predict for patient outcome in MBC. Our findings support the concept that circulating miRNAs represent non-invasive biomarkers with significant diagnostic and prognostic implications in breast cancer. Further studies in large homogenous populations using standardized techniques are required to establish the value of these markers.

## MATERIALS AND METHODS

### Patients

**Figure 6:**
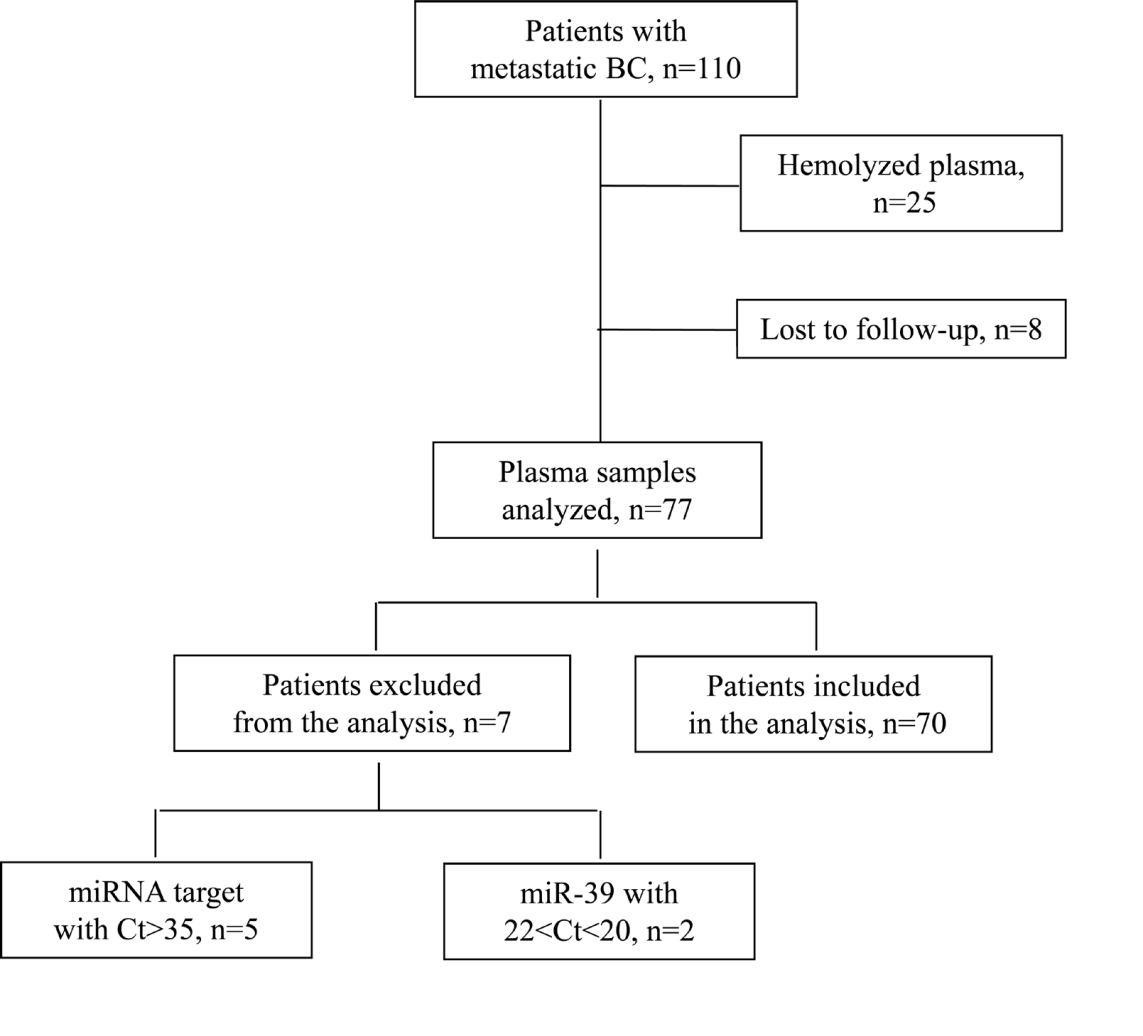
Flow chart of the study; Ct: cycle threshold

In the present study we included patients with early (*n* = 133) and metastatic (*n* = 110) breast cancer (Figure 6). The cohort of patients with early disease has been described in our previous work [24]. Patients with MBC were treated at the Department of Medical Oncology of the University Hospital of Heraklion (Crete, Greece) from 2003 – 2010. Peripheral blood samples were obtained before the initiation of first-line chemotherapy. Samples were also collected from 23 normal blood donors to serve as controls for miRNA evaluation. All patients and normal donors had signed an informed consent to participate in the study which was approved by the Ethics and Scientific Committee of the University Hospital of Heraklion, Greece.

Clinical characteristics and follow-up information for each patient were prospectively collected. Peripheral blood from healthy donors and patients was drawn early in the morning and was collected in EDTA- tubes. Plasma was subsequently isolated within 2 hours by centrifugation in 2500 rpm for 15 minutes at 4°C, followed by a second centrifugation in 2000 g for 15 minutes at 4°C, to remove cellular debris. Samples were kept in aliquots at −80° C until further use.

### RNA isolation

Total RNA was extracted from plasma using Trizol LS (Ambion, Life Technologies), as previously described [24]. Briefly, plasma was thawed on ice, centrifuged to remove cellular debris and 25 fmoles of the synthetic *C. elegans* miRNA cel-miR-39 (Qiagen Inc.-USA) was added after denaturation to each sample as an exogenous control. Aqueous phase was separated by adding chloroform followed by incubation on ice for 10 minutes. After centrifugation, equal volume of 700 μl of supernatant, from each sample containing the RNA was precipitated by adding 0.7 volumes of isopropanol and 1 μl of glycogen. RNA pellet was resuspended in 50 μl RNAse-free water. RNA from all samples was kept at −80° C until further use in the subsequent real-time qPCR.

Plasma samples presenting a change of colour to pink (*n* = 25), suggesting the presence of hemolysis and samples from patients lost to follow-up (*n* = 8), were not processed for RNA isolation (Figure 6).

### Quantitative real-time PCR analysis and miRNA expression

Reverse transcription and RT-qPCR was performed according to manufacturer’s instructions and as previously described [24]. In brief, total RNA input of 1.67 μl was reverse transcribed using the TaqMan miRNA Reverse Transcription kit and miRNA specific stem-loop primers (Applied Biosystmes, Foster City, CA, USA) in a 5 μl- reaction. cDNA was diluted at 30 μl and each miRNA was assessed by RT-qPCR. The quantitative real- time PCR reaction was carried out on a ViiA 7 Real- Time PCR System (Applied Biosystems, Foster City, CA, USA). All the assays were performed in triplicates. Appropriate negative controls were used in both cDNA synthesis and RT-qPCR reactions where RNA input was replaced by H_2_O and no template control was used, respectively. The average expression level for each miRNA was calculated by the 2^-ΔCt^ method relative to the average of miR-23a which was used as a reference gene. We choose miR-23a as an endogenous control since it was stably and reproducibly expressed among early and MBC patients and among patients and normal donors (Mann-Whitney test, *p* > 0.05). The fold change of target miRNAs relative to miRNA expressed in normal controls was calculated by the 2^-ΔΔCt^ method [55]. Samples with mean Ct>35 for target miRNAs (*n* = 5; Figure 6) as well as samples with mean Ct >22 or Ct <20 of cel-miR-39, suggestive of inefficient RNA extraction, were excluded from the analysis (*n* = 2; Figure 6). miR-451 and miR-23a expression levels were assessed to test for haemolysis in plasma samples, as previously described [56]. A total of 70 plasma samples were processed for further miRNA assessment (*n* = 70; Figure 6).

### Statistical analysis

The statistical analysis was performed using the SPSS software package, version 22.0 (SPSS Inc. Chicago IL). Patients were divided into high and low expression according to the median value for each miRNA expression. Patients with miRNA expression above or equal to the median values were characterized as having high, whereas those with miRNA expression below the median as having low expression. The median cut-off values were preserved in the whole group and across subgroup analysis. Correlations of expression between the different miRNAs were performed by Spearmans’ test. The chi-squared test was used to estimate associations between miRNA expression and clinicopathological characteristics. Mann-Whitney test was used to examine the differential expression between metastatic and early breast cancer patients. The associations between circulating miRNA expression levels and PFS or OS were assessed by Kaplan Meier method, log rank test (Mantel-Cox) and Cox proportional hazard regression models. PFS and OS were calculated from the start of treatment until the date of the first documented disease progression or death and last follow-up, respectively. To evaluate the value of circulating miRNAs in distinguishing between early and metastatic breast cancer, receiver operating characteristics (ROC) curves were constructed and areas under the curves (AUC) were calculated. The Youden index (sensitivity + specificity – 1) was used to set the optimal cut-off point. Statistical significance was set at *p* < 0.05 (two-sided test). This report is written according to the reporting recommendations for tumor marker prognostic studies (REMARK criteria) [57].

## Abbreviations

MBC: metastatic breast cancer
PFS: progression free survival
OS: overall survival
